# Adoption of Digital Mental Health Interventions in National Health Service England, Scotland, and Wales: Freedom of Information Questionnaire Study

**DOI:** 10.2196/92187

**Published:** 2026-05-14

**Authors:** Lucy Hitcham, Aislinn Gómez Bergin, Sachiyo Ito-Jaeger, Stuart Reeves, Elvira Perez Vallejos

**Affiliations:** 1 Horizon Centre for Doctoral Training University of Nottingham Nottingham, England United Kingdom; 2 Mental Health & Clinical Neuroscience, School of Medicine University of Nottingham Nottingham, England United Kingdom; 3 School of Computer Science University of Nottingham Nottingham, England United Kingdom; 4 NIHR MindTech HealthTech Research Centre Nottingham, England United Kingdom; 5 NIHR Nottingham Biomedical Research Centre Nottingham, England United Kingdom

**Keywords:** digital therapeutics, mental health services, digital, health care, National Health Service, United Kingdom

## Abstract

**Background:**

Digital mental health interventions (DMHIs) have been widely promoted to improve access to mental health care within the UK National Health Service (NHS), particularly following the COVID-19 pandemic. In 2015, a total of 48 technologies were reportedly used in NHS services in England, but over the past decade, substantial changes to regulatory requirements, evidence standards, and procurement processes have reshaped the digital mental health landscape. There is limited clarity regarding which DMHIs are currently being formally procured and funded by NHS mental health services across the United Kingdom.

**Objective:**

This study aimed to identify and describe the DMHIs currently procured, contracted, or paid for by NHS mental health service providers in England, Scotland, and Wales for adult common mental health problems and to compare current procurement practices with findings reported in 2015.

**Methods:**

Freedom of Information requests were submitted to all NHS mental health trusts in England and all health boards in Scotland and Wales. Responses were collated and screened to provide an updated and extended record of which technologies are reportedly procured or paid for by services.

**Results:**

In total, 19 different DMHIs were identified as being procured across mental health service providers for adult common mental health problems at the time of data collection. This demonstrates a substantial reduction in the number of technologies being adopted into practice compared to the 48 reported in England in 2015. The findings reveal several key insights, including that only 2 technologies have remained in use for a decade, and they shed light on the types of technologies being selected and the variations in procurement practices among the 3 national health services.

**Conclusions:**

Despite the expansion of the digital mental health marketplace, the number of DMHIs formally procured by NHS mental health services has markedly decreased over the past decade. This consolidation may reflect increased selectivity and the adoption of higher-quality products, driven by strengthened regulatory oversight, evidence standards, and national guidance. Although these developments may enhance safety and quality assurance, they also raise important questions about innovation, market sustainability, and equitable access to digital mental health care. Ongoing monitoring of procurement practices is needed to inform policy, service design, and the future development of DMHIs.

## Introduction

### Background

Globally, rates of mental health diagnoses, including depression and anxiety, are increasing [1,2]. In the United Kingdom, the delivery of mental health care for adult common mental health problems is primarily through the National Health Service (NHS) Talking Therapies (formerly known as Improving Access to Psychological Therapies). Referrals to this service are at an “all-time high,” with 1.82 million referrals in 2023 to 2024, compared to 884,000 received in 2012 to 2013 [3,4]. Additionally, services are facing other challenges, such as staff shortages, insufficient funding, long waiting lists, and care inequalities [5,6]. In response, wider access to interventions and support is needed. Digital mental health interventions (DMHIs) have been widely recommended as a potential solution for improving access to mental health care [7,8]. DMHIs encompass a wide range of different technologies, including smartphone apps, internet-based programs, extended and virtual reality (VR), wearable devices, and video games [8,9], aimed at supporting the management and prevention of mental health and well-being symptoms [10]. These interventions differ in delivery format (ie, clinician-supported, self-guided, or blended), therapeutic models (ie, cognitive behavioral, acceptance-based, or mindfulness-based), and digital complexity [11,12]. In line with service-level implementation, this study adopts a pragmatic definition of DMHIs that includes therapeutic programs and digitally enabled delivery platforms procured for mental health care. Their potential particularly lies in being able to offer more scalable, accessible, and cost-effective options for mental health care [9] and thus overcome barriers such as stigma, long waiting lists, and limited clinical time; they may also provide patients with greater choice in their treatment [8], potentially increasing empowerment and autonomy over their care. However, a greater choice of tools does not automatically mean that patients will be more empowered or autonomous. Access to digital mental health technologies is not always an indicator that people are accessing better or more effective support; therefore, the role of regulation and guidance in this space is crucial.

Many DMHIs are marketed directly to consumers (ie, via app stores); some are freely available, while others include costs such as subscriptions or in-app purchases. Within the NHS, several procurement models may be used to pay for and provide patients with access to DMHIs. However, although it is known that individual trusts procure certain DMHIs for their local service users, it is unclear which specific interventions are being used due to the lack of data availability and transparency, the lack of a national library from which they are selected, and fragmented, complex procurement pathways across services. There have been several studies aimed at understanding what DMHIs are available and evaluated [8,13,14]. These reviews often focus on young people, and it is valuable to understand what has been formally adopted into mental health services, rather than what is available in the literature. In 2015, Bennion et al [12] found that 48 different e-therapies were being used or recommended by the NHS for anxiety, depression, and stress. This was the first attempt to document what technologies were being used in practice in England, consisting of smartphone and web apps reported by Improving Access to Psychological Therapies services or listed in the NHS Health Apps Library. Since the publication of the study by Bennion et al [12] in 2015, no UK–wide empirical assessment has examined which DMHIs are formally procured by NHS services, making an updated review highly valuable.

Studies have reported that their use has become even more prolific in recent years due to the social distancing measures during the COVID-19 pandemic. Since 2015, there have been policy, regulation, and governance changes that indicate a shift toward greater provision of DMHIs. More broadly, recommendations in the NHS Long-Term Plans from 2019 and 2025 prioritize technology-enabled care [15,16]. Attempts at regulation and guidance have made considerable progress. Regulatory and health technology assessment agencies, such as the National Institute for Health and Care Excellence (NICE) and the Medicines and Healthcare products Regulatory Agency (MHRA), are important for ensuring that the risks of digital health technologies are mitigated and that they have the potential to improve care [7]. NICE issued its first guidance, which included DMHIs, in 2006 and currently provides guidance for depression and anxiety [17]. More recently, NICE has released its Evidence Standards Framework to guide NHS adopters in ensuring that DMHIs are effective and valuable and has begun conducting Early Value Assessments (EVAs) to support better evidence generation and adoption for health technologies in the NHS [18,19]. Within Scotland, the Scottish Health Technologies Group provides recommendations to NHS Scotland on health technologies. Likewise, Wales follows NICE guidance, but guidance is also issued by Health Technology Wales. Meanwhile, the Medicines and Healthcare products Regulatory Agency aims to legally regulate digital interventions that are classified as medical devices. Its most recent guidance, published in February 2025, provides information on whether DMHIs qualify as a software as a medical device and sets out the legal requirements for those that meet this threshold based on medical purpose and functionality [10]. Additionally, the NHS introduced the Digital Technology Assessment Criteria (DTAC) in February 2021 for technologies being used or procured by the NHS specifically. These criteria include aspects of clinical safety, data protection, usability, and accessibility to assess technologies [20].

NHS England has previously offered various iterations of a health app library, with the first commissioned in 2013 to provide patients with easier access to information and greater control over their care [21]. However, after concerns were raised about data security [22] and about the lack of an evidence base for the recommended apps [23], the library was suspended. Since then, several curated app libraries have appeared, which offer different levels of assessment, but many are no longer available online [23]. There is also considerable variation in how well equipped NHS trusts are to adopt technology-enabled care. The NHS Mental Health Implementation Plan emphasized the need for mental health service providers to reach a minimum level of digital maturity and the ambition for local services to offer a range of self-management apps, digital consultations, and digitally enabled therapies while supporting the development of new digital mental health technologies and online resources [24]. However, it is unclear whether these commitments have led to any meaningful changes in practice.

The expansion of these structures, regulatory frameworks, guidance, and commercial marketplaces has changed the landscape of digital mental health significantly over the last decade. Consequently, the use of DMHIs in the NHS has likely changed since the data collection by Bennion et al [12]. However, it remains unclear how these shifts have translated into the technologies that are formally adopted and sustained in mental health services. In this context, Freedom of Information (FOI) requests provide a uniquely appropriate method for examining real-world implementation, as they enable access to service-level information held by public bodies that is not observable through published literature, app libraries, or company claims. By focusing on technologies that are formally procured, contracted, or paid for by NHS providers, FOI methods allow the assessment of institutional adoption rather than availability, intention, or recommendation, offering a clearer picture of how digital mental health care is currently delivered within the NHS. Clarifying which digital tools are currently being used in mental health services for adult common mental health problems allows for an assessment of the impact of landscape changes and can highlight considerations for future development.

### Objectives

This study aimed to identify and characterize the DMHIs that are currently formally procured, contracted, or paid for by NHS mental health services in England, Scotland, and Wales for adult common mental health problems. In the United Kingdom, the FOI Act (2000) gives the public the right to request information from public authorities, including health care services, providing a helpful and underused way of collecting public data [25]. By using FOI requests to capture organizational-level procurement decisions, this study provides an empirical review of real-world digital mental health adoption within the NHS and examines how current patterns compare with those reported in England in 2015 by Bennion et al [12]. In doing so, the study seeks to inform understanding of how changes in regulation, evidence standards, and national guidance may be shaping the implementation and sustainability of DMHIs across UK health systems.

## Methods

### Design

This study adopted a cross-sectional descriptive design to examine organizational-level procurement of DMHIs. FOI requests were used to collect information on what DMHIs are being used in the NHS in England, Scotland, and Wales. The FOI questions reported in 2015 were used as a starting point for the design of this study [12]. These questions were updated to reflect current terminology through compiling literature and legislation and were vetted by several experts in the field. The FOI questions were amended to differentiate between technologies that are formally procured, contracted, or paid for by the NHS service provider and those being used or recommended within services to minimize ambiguity. These terms are defined as follows: “procured” was defined as formally purchased via a procurement process, “contracted” was defined as covered by a service contract or framework agreement, and “paid for” was defined as financial support or licensing without procurement ownership. Henceforth, we use the term procured to include all 3 terms. These questions were sent to FOI officers at a university and 2 NHS trusts to ensure their clarity and answerability before being finalized (refer to Multimedia Appendix 1 for questions sent via FOI requests). This study was expanded to include the health boards of Scotland and Wales to capture practice across the United Kingdom. Northern Ireland was not included due to significant differences in regulation and health care systems. FOI questions were tailored according to the inclusion and exclusion criteria (Textbox 1), and all reported technologies from the FOI requests were assessed against these criteria and screened.

The inclusion criteria for this study followed those of the 2015 study, which included e-therapies for adults targeting depression, anxiety, and stress that were locatable via a Google search. Listed technologies were excluded if they were intended for other conditions, such as eating disorders, sleep problems, and pain management; if they were unsearchable; or if they had more general health care functions, such as appointment scheduling, case management, or providing service information [12].

**Inclusion and exclusion criteria for reported technologies.**

**Inclusion criteria**

Technologies were included if they met the following criteria:

- Intended for adult services
- Relevant to common mental health problems (as defined by National Health Service Talking Therapies [4])
- Used for assessing risk, diagnosing, predicting, monitoring, treating, or preventing mental health conditions and/or symptoms [10]
- Identifiable through Google searches

**Exclusion criteria**

Technologies were excluded if they met any of the following criteria:

- Intended for child and adolescent services
- Relevant to serious mental health problems
- Intended for sleep problems
- Intended for neurodevelopmental disorders (eg, attention deficit hyperactivity disorder)

### Procedure

On September 5, 2024, an FOI request was sent to NHS England requesting a list of all current NHS mental health trusts and Talking Therapies providers in England. NHS England responded, advising that the details of mental health trusts are available in the provider directory [26], and a list of Talking Therapies providers can be found in the monthly and annual reports [4]. However, such reports do not include all Talking Therapy providers in England because NHS England does not hold this information. On this basis, Talking Therapies providers in England were not contacted directly, as NHS England does not maintain a comprehensive list of all Talking Therapy providers or their contact details, and responsibility for procurement typically sits at the trust level. Details of Scotland’s health boards were acquired from NHS Scotland [27], and the Welsh health boards were obtained from the Welsh Government website [17]. Compiled lists of trusts and health boards were checked against those listed by WhatDoTheyKnow, a website for generating and reporting previous FOI requests [28]. The contact details needed to submit FOI requests were obtained from each trust’s and health board’s website.

On December 12, 2024, a FOI request email was sent to each of the 50 mental health trusts in England, the 14 Scottish health boards, and the 7 Welsh health boards. Requests sought information on DMHIs that were currently procured, contracted, or paid for by the organization for adult common mental health problems (refer to Multimedia Appendix 1 for final questions). In line with the FOI Act 2000, all requests should be answered within 20 working days, and the request cannot exceed 18 hours of staff time to comply with cost limits [29]. Responses from all trusts and health boards were collated, and reported DMHIs were screened according to the inclusion criteria prior to analysis.

### Ethical Considerations

As this study used information obtained via FOI requests to public bodies and involved no collection of personal or patient-level data, formal ethics approval was not required as confirmed by the University of Nottingham Faculty of Medicine and Health Sciences ethics committee (application ID FMHS 301-0924). All FOI responses and the collated data are available via the Open Science Framework repository (refer to the Data Availability section for details)*.*

## Results

### Overview

**Figure 1:**
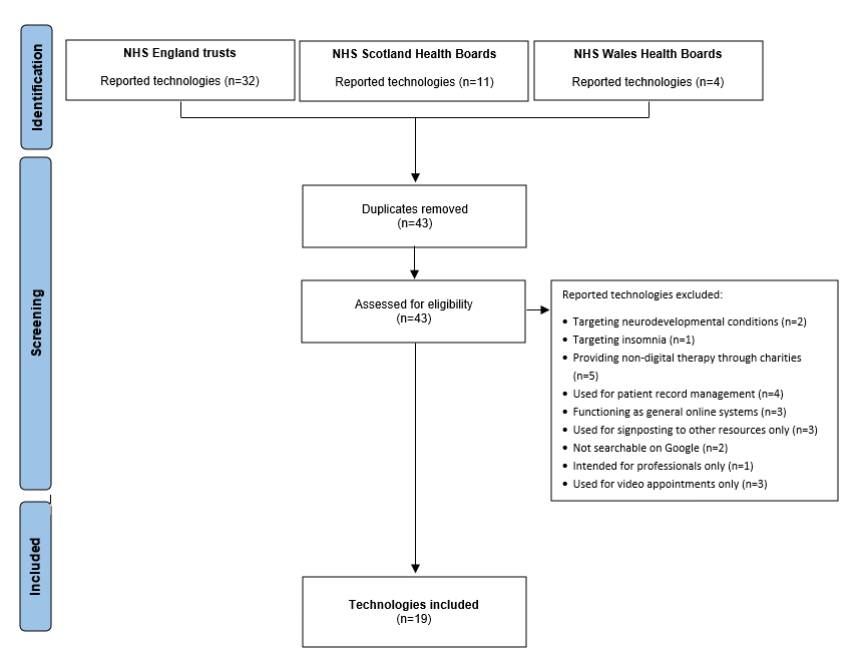
Flow diagram of the screening process for reported technologies.

We present the findings of the FOI requests, including the DMHIs procured or used by the NHS mental health trusts in England and the Scottish and Welsh health boards. Overall, the findings indicate a relatively small and concentrated set of DMHIs being formally procured across NHS services, with variation in procurement strategies across England, Scotland, and Wales. We provide details on the response rates and the technologies reported as procured, including any technologies reported as being piloted or previously used, by the different NHS services. The final list of DMHIs reported as being procured, contracted, or paid for by trusts or health boards in England, Scotland, and Wales for adult common mental health problems can be found in Table 1 [30-48]. The screening process for technologies that met the inclusion criteria can be found in Figure 1, and a list of all excluded technologies is available in the Open Science Framework repository.

**Table 1:** List of digital mental health interventions procured by services in England, Scotland, and Wales.

Name of intervention	Type of technology	Number of National Health Service providers procuring the technology, n (%)		
		England (n=47 trusts)	Scotland (n=12 health boards)	Wales (n=7 health boards)
SilverCloud [32]	Online CBT^a^ modular self-help course	32 (68)	12 (100)	3 (42.9)
IESO [33]	Online 1:1 messenger-based therapy with a professional	13 (27.7)	12 (100)	0 (0)
Daylight [34]	CBT self-help program for anxiety	0 (0)	12 (100)	0 (0)
Limbic [35]	AI^b^-based triage assistant	6 (12.7)	0 (0)	0 (0)
Wysa [36]	AI-based app with CBT programs	4 (8.5)	0 (0)	0 (0)
Xyla [37]	Guided digitally enabled therapy	9 (19.1)	1 (8.3)	0 (0)
Dr Julian [38]	Video consultation platform	3 (6.3)	0 (0)	0 (0)
Koa Health [39]	Internet-delivered cognitive therapy	2 (4.2)	0 (0)	0 (0)
AccuRx [40]	Video consultation platform	2 (4.2)	0 (0)	0 (0)
HelloSelf [41]	Video consultation platform	2 (4.2)	0 (0)	0 (0)
Bilateral Base [42]	Online EMDR^c^ therapy	1 (2.1)	0 (0)	0 (0)
Omnitherapy [43]	Online video courses for different mental health problems	1 (2.1)	0 (0)	0 (0)
Monsenso^d^ [44]	Remote monitoring and treatment support using patient data	1 (2.1)	0 (0)	0 (0)
VR Headset by XRHealth [45]	Virtual reality headset	1 (2.1)	0 (0)	0 (0)
Recap Health [46]	Resource sharing platform	1 (2.1)	0 (0)	0 (0)
Calm Distress [47]	Online modular self-help course	0 (0)	1 (8.3)	0 (0)
CloudEMDR [48]	Online EMDR therapy	0 (0)	1 (8.3)	0 (0)
Serenity [49]	Online CBT modular self-help course	0 (0)	0 (0)	1 (14.2)
Livi [50]	Video consultation platform	1 (2.1)	0 (0)	0 (0)

^a^CBT: cognitive behavioral therapy.

^b^AI: artificial intelligence.

^c^EMDR: eye movement desensitization and reprocessing.

^d^Pilot study.

### NHS England Trusts

All 50 NHS mental health trusts in England responded to the FOI request. One trust declined to respond, citing cybersecurity concerns, and another 2 trusts stated that gathering the information would take longer than the 18-hour time allowance for FOI requests. This left a total of 47 responses to report. Overall, 7 (15.2%) of the 47 NHS trusts reported that they were not procuring, contracting, or paying for digital mental health technologies. The remaining 40 (85.1%) trusts reported 32 technologies and other digital resources, 17 (53.1%) of which did not meet the inclusion criteria (Figure 1). This left 15 (31.9%) DMHIs included (Table 1). Of the 48 apps reported in 2015, only SilverCloud and IESO were still reportedly procured, with SilverCloud being the most widely procured across NHS England. One trust reported a pilot of Monsenso’s technology, while another was uniquely procuring immersive technology in the form of VR headsets provided by XR Health. Two trusts indicated that they previously procured MindDistrict, but this technology is no longer on the market. Additionally, 2 trusts reported in-house developments. One had developed a bespoke e-clinic app to deliver therapy by synchronous messaging, and another had developed self-help workbooks. However, neither had a website to confirm their eligibility for inclusion. Procurement in England was characterized by a relatively wide range of technologies, procured independently by individual trusts.

### NHS Scotland

All 14 health boards in Scotland responded to the FOI request. One health board declined to respond, citing cybersecurity concerns. This left a total of 13 (92.8%) responses to report. The health boards reported 11 procured technologies, 5 (45%) of which did not meet the inclusion criteria. This left 6 (55%) DMHIs to report (Table 1). In total, 3 (27.3%) of the included technologies were reportedly procured centrally by the Scottish Government for use across all health boards as part of Scotland’s Digital Mental Health Programme [49]. Therefore, although some health boards reported not directly procuring DMHIs, many did list the technologies they had procured as part of this program. Some of the health boards also reported the use of additional technologies beyond those nationally procured. One health board was piloting the use of Xyla; another had procured technology for eye movement desensitization and reprocessing therapy, provided by CloudEMDR; and another had 2 DMHIs developed in-house: Calm Distress and Lanarkshire Mind Matters. In contrast to England, several DMHIs were centrally procured for use across Scottish health boards, resulting in greater uniformity in reported platforms.

### NHS Wales

All 7 Welsh health boards responded to the FOI request. Of these, 2 (28.6%) health boards reported procuring no technologies. The remaining 5 (71.4%) health boards named 4 different technologies, 2 (50%) of which did not meet the inclusion criteria (Figure 1). This left 2 (50%) DMHIs to report (Table 1). Overall, fewer DMHIs were reported across Welsh health boards, with limited overlap between service providers.

## Discussion

### Principal Findings

This study provides a current review of the DMHIs that are being procured by health services in the United Kingdom. Within England, of the 50 trusts contacted, 3 declined to respond, and 7 reported procuring no DMHIs. The final 40 trusts reported using 32 technologies, of which 15 were eligible for inclusion. Within Wales, all health boards responded to our FOI request, and 2 reported no DMHIs. Of the remaining 5, they reported using 4 different DMHIs, of which 2 were eligible. In Scotland, 1 health board declined to provide the requested information, while the remaining health boards reported using DMHIs, with a total of 11, 6 of which were eligible for inclusion. After considering duplication of use or procurement, a total of 19 DMHIs were identified across the United Kingdom.

This study demonstrates a marked consolidation in the DMHIs formally procured by NHS services, despite significant growth in the digital mental health marketplace over the last decade. As a direct comparison, because the study by Bennion et al [12] was only conducted within England, the number of DMHIs used within trusts has dropped from 48 to only 15. Across England, Scotland, and Wales, this increases to only 19, less than half the number available to staff and patients in 2015. Of those reported, only SilverCloud and IESO have remained in use and operational across this time. Although the technologies in 2015 were predominantly web-based platforms and smartphone apps, current procurement includes a wider set of mental health technologies, including an online eye movement desensitization and reprocessing platform and a VR headset. This shift in the types of technologies being procured aligns with wider developments in the marketplace and research, reflecting the increasingly diverse options available. We also highlight variation in adoption strategies across the 3 countries. Greater uniformity is found in Scotland due to a more centralized national procurement approach, compared with the more fragmented, local-level adoption in England and Wales, reflecting how governance and commissioning structures may play a substantial role in shaping digital mental health implementation. This decentralized and uncoordinated landscape makes it difficult to achieve consistency in adoption, limits visibility of which technologies are in use, and creates substantial barriers for both NHS service providers and technology developers seeking to implement or scale DMHIs. Likewise, it determines which technologies patients are or are not able to access, significantly impacting the choice and autonomy they have in their care. This stands in contrast to Scotland, where greater uniformity is achieved through a centralized program, which may facilitate clearer pathways to adoption, reduce duplication of effort, and improve transparency regarding which technologies are being used in practice. However, the implementation of a centralized procurement program in England would be a complex, long-term process change.

Although responsibility for the procurement of these technologies primarily lies at the trust level, it is possible that a smaller number of DMHIs have been reported compared to the study by Bennion et al [12] if there is a higher rate of use among Talking Therapies services. However, this would suggest that Talking Therapy services are independently procuring and using DMHIs that are not being shared with trusts. Importantly, our study captures institutional adoption through formal procurement, rather than informal recommendation, short-term pilots, or in-house developments. However, this may also reflect market maturity rather than a reduction in innovative activity within the NHS. This may account for some of the reduction in reported technologies, but many trusts and health boards were unable to report recommended DMHIs accurately due to variation between clinicians and patient needs. However, this consolidation of procured tools may signal that only higher-quality, safer, and more effective technologies are being procured by services following changes to regulation and guidance.

### The Regulation of DMHIs

One of the most prominent shifts over the last decade has been changes to evidence standards, regulatory oversight, assessment criteria, and procurement guidance for DMHIs. The reduction in procured technologies likely reflects these shifts, rather than declining interest in digital mental health, resulting in a more evidence-based and compliance-focused approach to adoption. However, this may have trade-offs when it comes to innovation, access, and equity.

The demise of NHS-led health app libraries has made way for commercially curated app libraries, and the NHS has instead turned to assessments such as the DTAC. NICE guidance has been updated, and new approaches, such as the EVAs, have been adopted. These changes may have created an unstable marketplace for manufacturers and users alike, but they have also led to important progress in establishing the standards for evidence generation, data security and protection, clinical safety, and value proposition. All mental health interventions being adopted into the NHS now require approval under the DTAC, which aligns with the Evidence Standards Framework and General Data Protection Regulation requirements, as well as a digitally enabled therapy assessment from NHS England. These tools are also assessed for whether they qualify as software as a medical device and may therefore require Conformité Européenne marking or UK Conformity Assessed approval. This suggests that there is a higher expected standard for DMHIs adopted within the NHS, potentially bringing more safety and assurance to both clinical professionals and service users. This may account for the reduction in DMHIs being adopted within the NHS. We suggest that this indicates the success of these regulations and standards and that it may be the most effective, acceptable, and safe DMHIs being implemented into services for patients. However, we recognize that the lack of direct responses from Talking Therapy services may mean that the requests did not capture all DMHIs that are in use across all services. The websites of DMHIs not included in this list often claim adoption by various NHS services, even though those services did not confirm this in our request.

Another area to consider is whether these higher standards may stifle innovation and digital adoption within mental health services. There are many possible advantages of implementing DMHIs into care, such as freeing up clinical time, addressing access barriers, offering data-driven and personalized care, and providing patients with greater choice and control over their care [3,8]. Potentially, stricter regulation could impede the innovation and adoption process, leading to a situation where companies are unable to remain viable long enough to develop and deliver high-quality interventions. They may also be more likely to rely on less stable sources of investment, such as grant funding. Although it could be argued that mental health should not be a “start-up” space, it is undeniable that we need more choice and access for patients and services. Although initiatives such as the NICE EVA aim to identify and support interventions that offer promise and value to care [19], this is further complicated by other barriers to innovation and adoption into the NHS. For instance, small and medium-sized enterprises may struggle to meet the expectations of evidence generation and large-scale contracts [50], which often take several years and significant funding. The NHS’s resistance to change and lack of resources are compounded by differing views on what constitutes important evidence. Consequently, trust board members often feel inadequately prepared to assess this evidence and make decisions about technology procurement [51], despite many NHS trusts having in-house development teams that work directly with clinicians [52]. Faced with these barriers, our results may simply indicate that manufacturers are instead opting for direct-to-consumer routes. Therefore, although it is vital that technologies are proven to be effective and safe for users, there may be a balance to be struck that avoids stifling innovation while still allowing promising DMHIs the economic stability needed to achieve successful implementation and scale. The NHS 10-Year Plan [15] has laid out an ambitious plan for the NHS, particularly within the area of artificial intelligence adoption in services. Within this plan, an innovator passport will be introduced so that when one NHS organization has assessed an innovation, this will then be available to other organizations without requiring repeated assessments. A new digital marketplace will be established to procure technologies. What these will look like in practice is so far unsure, but importantly, these herald a shift toward speedier adoption of innovation in health care, which is likely to increase the number of DMHIs procured. Funding, resources, and initiatives to promote innovation are important, and future research could aim to understand this from the perspective of developers. As digital mental health continues to be positioned as a key component of mental health service transformation, ongoing monitoring of procurement and adoption will also be essential to ensure that regulatory standards, innovation, and patient access remain appropriately aligned.

### Limitations

This study relied heavily on FOI requests for data collection. FOI requests can be a powerful tool for researchers to obtain data held by public bodies, such as the NHS, and to contextualize or complement research, but they are often underused [25]. In this study, they allowed us to document what is happening in practice in the United Kingdom–wide mental health services for the first time, which could not have been investigated with other research methods. Future researchers could consider using FOI requests to collect other mental health data, extending this approach to child services, for example, or other countries where applicable. However, these data are limited by the interpretation of the person replying to the request and whether the information is readily available. Our findings echo those of Bennion et al [12], reaffirming that information about the use of DMHIs across NHS trusts remains difficult to access. Like the original study, we found that much of this information is not publicly available and requires formal requests to obtain. This may also lead to possible gaps in the data, such as technologies that may be procured by services but were not reported in the received responses. This lack of visibility limits opportunities for shared learning, cross-trust collaboration, and the development of consistent, evidence-based digital care pathways. Making this information more readily accessible would not only support service improvement and innovation but also help ensure equitable access to effective digital mental health support across the NHS. This is highlighted by the absence of a central database for all Talking Therapies providers, which suggests that this study may not fully account for all mental health services in England. Additionally, the 4 trusts and health boards that declined to provide information represent a limitation through missing data, and the data collected are only accurate at the time of answering and can rapidly become outdated.

### Conclusions

This study shows a substantial consolidation in the DMHIs formally procured by NHS services across England, Scotland, and Wales over the past decade. Only 2 of the 48 technologies reported in 2015 remain in use, underscoring a marked reduction in the number and diversity of procured DMHIs. These findings suggest that increasing regulatory oversight and evidence standards are potentially shaping procurement choices, favoring a smaller set of higher-quality, safer interventions. However, regulation must be carefully balanced with the need to foster innovation and enable the safe adoption of novel tools. Looking ahead, continued monitoring of procurement patterns will be essential to ensure that health systems remain responsive to emerging technologies. However, there is a need to improve the ease of access and visibility of these data, while future research and technology developers should focus on ensuring the safety and effectiveness of these technologies. For policymakers and NHS leaders, these results underscore the need to align procurement strategies with both regulatory frameworks and innovation pathways, while recognizing the dynamic nature of digital innovation in mental health.

## Appendix

Multimedia Appendix 1 Freedom of Information request questions sent to National Health Service trusts and health boards.

## Abbreviations

DMHI: digital mental health intervention
DTAC: Digital Technology Assessment Criteria
EVA: Early Value Assessment
FOI: Freedom of Information
NHS: National Health Service
NICE: National Institute for Health and Care Excellence
VR: virtual reality

## Data Availability

All data collected and analyzed as part of this study are openly available via the Open Science Framework repository [53]. This repository includes collated Freedom of Information (FOI) responses, extracted datasets, and supporting documentation used in the analysis. Individual FOI responses were obtained from National Health Service trusts and health boards and are reproduced as provided at the time of response.
