# Supplementary material for: Adoption of Digital Mental Health Interventions in National Health Service England, Scotland, and Wales: Freedom of Information Questionnaire Study
Source: JMIR Ment Health. 2026 May 14;13:e92187. doi: 10.2196/92187 (PMC13219984; doi:10.2196/92187)
Supplement: Multimedia Appendix 1 [file mental_v13i1e92187_app1.pdf]

Supplementary material

Freedom of Information request questions for Mental Health Trusts of NHS England

1. Please state any digital technologies, e-therapies, internet-delivered therapies, online or digital therapies, digitally enabled therapies or digital therapeutics for adult mental health problems that your Trust has procured, contracted or are paid for by the service for use by service users. For example, these might include, but are not limited to, internet-delivered cognitive behaviour (CBT) with or without therapist support, smartphone applications, web applications or programmes, wearables (including devices or sensors) or extended reality technologies (i.e. virtual reality, augmented reality). Format this as a list indicating which have been procured, contracted or paid for by the service.

2. Additionally, please state any digital technologies, e-therapies, internet-delivered therapies, online or digital therapies, digitally enabled therapies or digital therapeutics for adult mental health problems that your Trust uses or recommends to service users. For example, these might include, but are not limited to, internet-delivered cognitive behaviour (CBT) with or without therapist support, smartphone applications, web applications or programmes, wearables (including devices or sensors) or extended reality technologies (i.e. virtual reality, augmented reality). Format this as a list indicating which are recommended and which are used by the Trust.

Please note: we distinguish between 1) procured, contracted or paid for by the service and 2) used and recommended based on the following:

1) *Procured, contracted or paid for by the service* - these are technologies from suppliers that have been formally agreed and paid for by the Trust to use in service.
2) *Used or recommended* - these are technologies that have not been formally procured or contracted but might be suggested to service users (e.g. a list of technologies given upon referral, while on the waitlist for treatment, upon treatment commencement or completion) or used by clinicians in service.

3. For the period of 2023/24, please provide any data monitoring information you collect on the technologies listed above. For example, the number of people that have been recommended or reported using the technologies, how long they continued to use the technology or whether there was a reported benefit from their use. Please state where this data is collected from (e.g. the technology providers or from the service).

4. Please list any Talking Therapies (formerly known as IAPT) providers that run services on your behalf or in connection with your Trust.

5. If these services are not run directly by the NHS please state who runs them and what they are (e.g. Social Enterprise, Limited Company, third sector group).

6. Please state any organisations or third parties (e.g. ORCHA) you have partnered with or commissioned to create or provide an app library.

7. For the period 2023/24 please state the total number of people your Trust treated for common mental health problems (see definition: here), including how many were treated for each problem.

Freedom of Information request questions for Health Boards of NHS Scotland and Wales

1. Please state any digital technologies, e-therapies, internet-delivered therapies, online or digital therapies, digitally enabled therapies or digital therapeutics for adult mental health problems that your Health Board service have been procured, contracted or are paid for by the service for use by service users. For example, these might include, but are not limited to, internet-delivered cognitive behaviour (CBT) with or without therapist support, smartphone applications, web applications or programmes, wearables (including devices or sensors) or extended reality technologies (i.e. virtual reality, augmented reality). Please format this as a list indicating which have been procured, contracted or paid for by the service.

2. Additionally, please state any digital technologies, e-therapies, internet-delivered therapies, online or digital therapies, digitally enabled therapies or digital therapeutics for adult mental health problems that your Health Board service uses or recommends to service users. For example, these might include, but are not limited to, internet-delivered cognitive behaviour (CBT) with or without therapist support, smartphone applications, web applications or programmes, wearables (including devices or sensors) or extended reality technologies (i.e. virtual reality, augmented reality). Please format this as a list indicating which are recommended and which are used by the Health Board.

> *Please note:* we distinguish between 1) used and recommended and 2) procured, contracted or paid for by the service based on the following:
>
> a. *Procured, contracted or paid for by the service* - these are technologies from suppliers that have been formally agreed and paid for by the Health Board to use in service.
> b. *Used or recommended* - these are technologies that have not been formally procured or contracted but might be suggested to service users (e.g. a list of technologies given upon referral, while on the waitlist for treatment, upon treatment commencement or completion) or used by clinicians in service.

3. For the period of 2023/24, please provide any data monitoring information you collect on the technologies listed above. For example, the number of people that have been recommended or reported using the technologies, how long they continued to use the technology or whether there was a reported benefit from their use. Please state where this data is collected from (e.g. the technology providers or from the service).

4. Please list any separate Community Mental Health Teams (CMHTs) that run services on your behalf or in connection with your Health Board.

5. Please state any organisations or third parties (e.g. ORCHA) you may have partnered with or commissioned to create or provide an App Library.

6. For the period of 2023/24, please state the total number of people your Health Board treated for common mental health problems, including how many were treated for each problem.
